# Gentiopicroside modulates glucose homeostasis in high-fat-diet and streptozotocin-induced type 2 diabetic mice

**DOI:** 10.3389/fphar.2023.1172360

**Published:** 2023-08-04

**Authors:** Xing Wang, Dongmei Long, Xianghong Hu, Nan Guo

**Affiliations:** ^1^ Department of Pharmacology, School of Pharmacy, North Sichuan Medical College, Nanchong, China; ^2^ Nanchong Key Laboratory of Disease Prevention, Control and Detection in Livestock and Poultry, Nanchong Vocational and Technical College, Nanchong, China; ^3^ Department of Pharmacy, Minhang Hospital, Fudan University, Shanghai, China

**Keywords:** gentiopicroside, type 2 diabetes mellitus, hepatic gluconeogenesis, PI3K/akt pathway, FOxO1

## Abstract

Gluconeogenesis is closely related to the occurrence and development of type 2 diabetes mellitus (T2DM). Gentiopicroside (GPS) is the main active secoiridoid glycoside in Gentiana manshurica Kitagawa, which can improve chronic complications associated with diabetes and regulate glucose metabolism. However, the effects and potential mechanisms by which GPS affects T2DM understudied and poorly understood. In this study, we systematically explored the pharmacological effects of GPS on T2DM induced by a high-fat diet (HFD) and streptozotocin (STZ) as well as explored its related mechanisms. The results showed that GPS supplementation discernibly decreased blood glucose levels, food intake and water consumption, ameliorated glucose intolerance, abnormal pyruvate tolerance, insulin resistance and dyslipidemia. Furthermore, GPS discernibly ameliorated pathological morphological abnormalities of the liver and pancreas, reduced hepatic steatosis and maintain the balance between α-cells and β-cells in pancreas. Moreover, GPS significantly inhibited gluconeogenesis, as evidenced by the suppressed protein expression of phosphoenolpyruvate carboxykinase (PEPCK) and glucose 6-phosphatase (G6Pase) in the liver. Additionally, the results of Western blot analysis revealed that GPS increased p-PI3K, p-AKT, and p-FOXO1 expression levels, and decreased FOXO1 expression at protein level in the liver. Furthermore, the results of the immunostaining and Western blot analysis demonstrated that GPS supplementation increased the expression of zonula occludens-1 (ZO-1) and occludin in the ileum. Collectively, these results indicate that GPS may inhibit hepatic gluconeogenesis by regulating the PI3K/AKT/FOXO1 signaling pathway and maintain intestinal barrier integrity, and ultimately improve T2DM. Together, these findings indicate that GPS is a potential candidate drug for the prevention and treatment of T2DM, and the results of our study will provide experimental basis for further exploration of the possibility of GPS as a therapeutic agent for T2DM.

## 1 Introduction

Owing to rapid urbanization, the aging population, as well as lifestyle changes and improvements in society, which has resulted in an increase in the number of diabetic patients annually. More than 90% of diabetes patients are diagnosed with type 2 diabetes mellitus (T2DM) (Laakso et al., 2022), which is a chronic metabolic disease characterized by hyperglycemia, intestinal flora disorder, insulin resistance, and obesity due to insufficient insulin secretion and an ineffective insulin response, and has become disease that has caused a high public burden worldwide (Ahmad et al., 2022; Magliano et al., 2022). According to the 10th edition of the IDF Diabetes Atlas, the number of diabetic patients was 537 million worldwide in 2021. This number is estimated to rise to 643 million by 2030 and 784 million by 2045 (Sun et al., 2022). When blood glucose levels are not well regulated, it can gradually give rise to more serious chronic microvascular or macrovascular complications, which severely affect people’s quality of life and create a huge economic burden (Yingrui et al., 2022). Therefore, it is essential to identify novel therapeutic strategies and develop effective drugs that can inhibit the progression of T2DM.

Under normal circumstances, the body maintains its blood glucose level by regulating the balance between the production and absorption of glucose. The liver is one of three major target organs that is affected by insulin and plays a fundamental role in maintaining body’s blood glucose homeostasis (Liang et al., 2022). The maintenance of glucose balance in the body depends on glucose uptake by the muscles and adipose tissues, as well as glucose production by gluconeogenesis and glycogenolysis in the liver (Yan et al., 2019). The liver mainly regulates blood glucose levels by regulating hepatic glucose production (HGP), which is mainly the result of gluconeogenesis and liver glycogen breakdown, while 90% of endogenous glucose production is mediated by HGP (Fujimoto et al., 2022). The abnormal modulate of glucose production in the liver is the principal cause of hyperglycemia and insulin resistance in T2DM (Giralt et al., 2018). Notably, in the diabetic state, excessive HGP exceeds systemic glucose uptake, which in turn leads to the dysregulation of blood glucose levels. Moreover, during diabetes, gluconeogenesis levels in the liver are abnormally high and are accompanied by high levels of cholesterol and triglyceride synthesis, which eventually develops into hyperglycemia and hyperlipidemia if not controlled in time (Tseng et al., 2019). In addition, many studies have shown that HGP inhibition is an effective strategy used for the prevention and treatment of T2DM (Liu et al., 2015; Wu et al., 2021). Therefore, gluconeogenesis inhibition should be an effectual strategy for the prevention and treatment of T2DM.

Gentiopicroside (GPS) is an iridoid glycoside isolated from the perennial herbs of Gentianaceae and is widely used in China as a herbal medicine for the treatment of rheumatoid arthritis, hemiplegia, joint pain, stroke, and hypertension (Xing et al., 2021). In addition, GPS has been found to exert anti-inflammatory, anti-oxidant, and cholagogic pharmacological activities in all kinds of disease models (Wu et al., 2017; Li et al., 2018; Jin et al., 2020). In regard to diabetes and its complications, GPS significantly improved peripheral neuropathy in STZ-induced diabetic rats, and its effects may be associated with the improvement of neural blood flow and the regulation of dyslipidemia (Lu et al., 2018). GPS significantly improved diabetic retinopathy and diabetic nephropathy by inhibiting the inflammatory response and oxidative stress (Zhang et al., 2019; Xu et al., 2022). GPS activated the PI3K/AKT signaling pathway by targeting progestin and adipoQ receptor 3 (PAQR3), thereby significantly improving glycolipid metabolism disorder in high-fat fed mice (Xiao et al., 2022). GPS has been found to maintain glucose homeostasis by inhibiting gluconeogenesis in L02 cells and mice (Yang et al., 2018). In addition, GPS improved atherosclerosis in high-fat diet-induced ApoE^−/−^ mice by modulating intestinal flora and fecal metabolites (Xie et al., 2022). Although many studies have shown that GPS can improve symptoms of hyperglycemia in diabetes, but it remains to be ascertained whether its effects are associated with gluconeogenesis and intestinal permeability. Based on the aforementioned analysis, we further investigated whether GPS improves T2DM by inhibiting gluconeogenesis and regulating the intestinal permeability.

## 2 Materials and methods

### 2.1 Reagents and antibodies

Gentiopicroside (C_16_H_20_O_9_, MW: 356.3, purity: ≧ 98%) and STZ (purity≧99%) were obtained from Yuanye Bio-Technology Co. Ltd (Shanghai, China). The kits used to determine triglyceride (TG), total cholesterol (TC), high-density lipoprotein cholesterol (HDL-C), and low-density lipoprotein cholesterol (LDL-C) levels were obtained from Biosino Biotechnology and Science Inc (Beijing, China). The kits for assessing alanine transaminase (ALT) and aspartate transaminase (AST) activities were purchased from the Nanjing Jiancheng Bioengineering Institute (Nanjing, China). The kit used to determine the glycosylated hemoglobin (HbA1c) level was purchased from Beijing Homa Biological Engineering Co. Ltd (Beijing, China), while kit for insulin detection was obtained from CAMILO BIOLOGICAL (Nanjing, China). Antibodies against phosphoenolpyruvate carboxylase (PEPCK), glucose 6-phosphatase (G6Pase), zonula occludens-1 (ZO-1), and occludin were purchased from Affinity Bioscience (United States). Antibodies against phosphoinositide 3-kinase (PI3K), protein kinase B (AKT) and β-actin were obtained from Wuhan Servicebio Technology Co. Ltd (Wuhan, China). Antibodies against forkhead box O1 (FOXO1), phospho-PI3K (Tyr458), phospho-AKT (Ser473), phospho-FOXO1 (Ser256) were bought from Cell Signaling Technology (Danvers, MA, United States). The Polyvinylidene difluoride (PVDF) membranes was bought from Millipore (CA, United States). Bicinchoninic acid (BCA) protein kit, RIPA lysis buffer, protease and phosphatase inhibitors, enhanced chemiluminescence (ECL) reagent, and the horseradish peroxidase (HRP)-labeled IgG were obtained from Applygen Technologies (Beijing, China).

### 2.2 Animals

Forty male C57BL/6 J mice (aged 8 weeks old; weighing 20–22 g) were purchased from HFK Bioscience (Beijing, China). The mice were kept in a room at a relative humidity of 55% ± 10%, a temperature of 23°C ± 2°C, and a light/dark cycle of 12 h. The mice were allowed free access to drinking water and standard laboratory food. Animals and all study protocols were performed strictly in accordance with Chinese standards and guidelines for the use of laboratory animals (GB14925-2001 and MOST 2006 a), and were carried out with the approval of the Experimental Animal Welfare Ethics Committee of the North Sichuan Medical College (Approval No. 202205).

### 2.3 Experimental design

**FIGURE 1:**
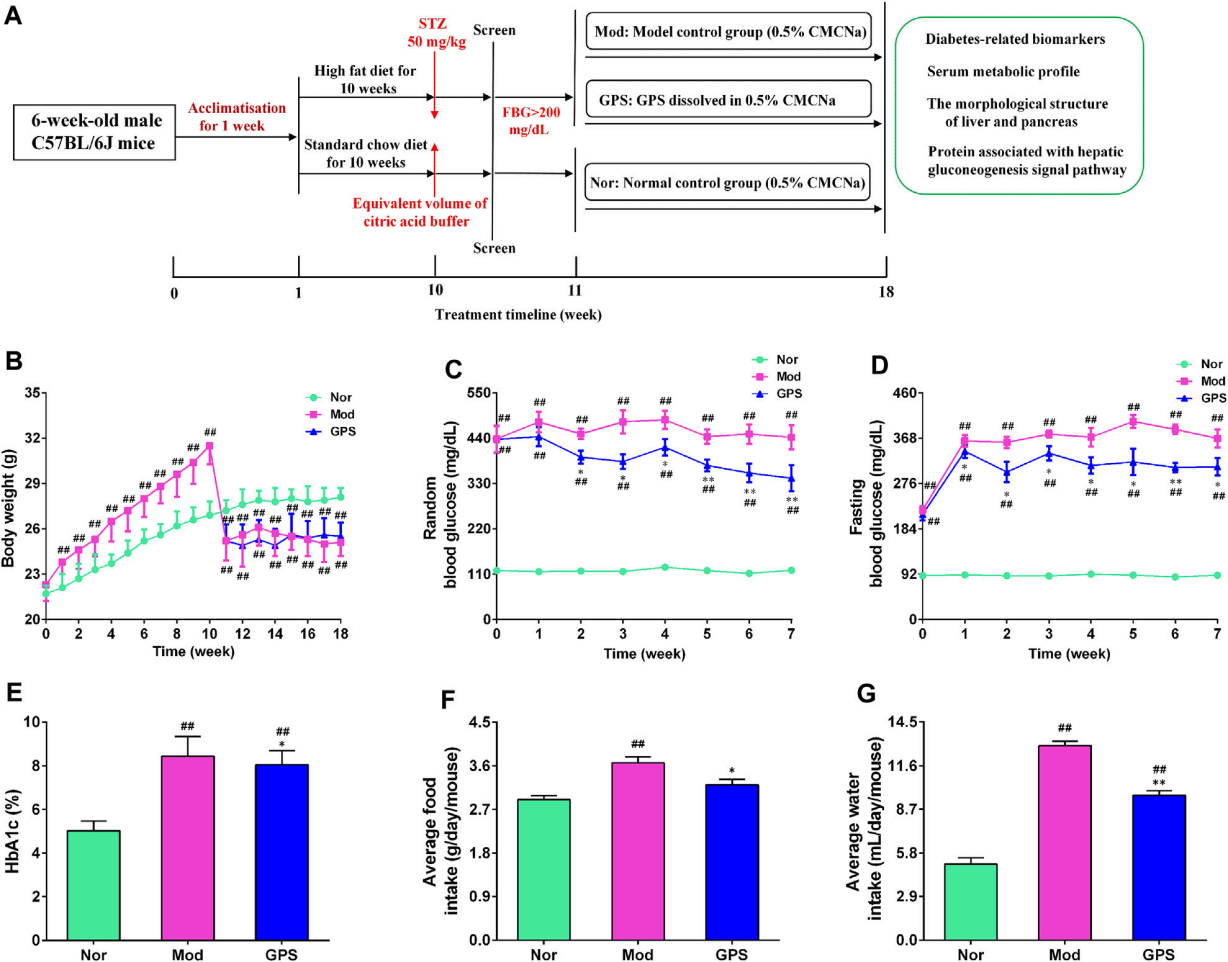
GPS attenuated metabolic disorders in HFD and STZ-induced diabetic mice **(A)** The flowchart of treatments administered to the mice **(B)** Body weight **(C)** RBG concentrations **(D)** FBG concentration **(E)** HbA1c **(F)** Average food intake **(G)** Average water concentrations. Data are presented as means ± SEM, *n* = 10. ^*#*^ *p* < 0.05; ^*##*^ *p* < 0.01 vs. Nor group; *p < 0.05; **p < 0.01 vs. Mod group.

The establishment of T2DM mouse model referred to the following literature with a small change (Yan et al., 2018; Zhang et al., 2018; Xiao et al., 2022; Xie et al., 2022). The 30 mice were adaptively fed for a week, and were then administered a high-fat diet (HFD, 60% fat), while the other 10 mice were fed on a normal diet for 8 weeks. Subsequently, the HFD mice were fasted for 6 h and injected with STZ (50 mg/kg) dissolved in an ice-cold citrate buffer (0.1 M, pH 4.5), on 4 consecutive days. The mice that were given a normal diet were intraperitoneally injected with a citric acid buffer. After 3 days, the fasting blood glucose (FBG) level of each mouse was measured, and mice with FBG >200 mg/dL were regarded as diabetic mice and were selected for further study. The diabetic mice were divided into two groups, based on their blood glucose level and body weight: 1) the diabetic model group (Mod); and 2) the GPS intragastric administration treatment group (GPS, 50 mg/kg). The dosage selected was based on previously published articles (Lu et al., 2018; Xiao et al., 2020; Xiao et al., 2022; Xu et al., 2022; Zou et al., 2022). Mice that were administered a normal diet were used as the normal control group (Nor). After the mice were divided into groups, GPS was dissolved in 0.5% sodium carboxymethyl cellulose (CMC-Na) to create a suspension, and the suspension was administered through gavage for 7 weeks, once a day. The mice in the Nor and Mod groups were orally administered a 0.5% CMC-Na suspension. Then, random blood glucose (RBG), FBG, and body weight were measured weekly during the experiment. After 7 weeks of treatment, the mice were fasted for 6 h, and after being administered pentobarbital sodium anesthesia, whole blood samples were collected through retroorbital puncture and were allowed to stand at room temperature for 2 h. Then, serum was collected through centrifugation at 4°C and 6,000 g for 10 min, and frozen until the analysis was conducted. The liver, pancreas and small intestine were immediately removed, and a few of these samples were fixed in 4% neutral paraformaldehyde for histological analysis, while the rest were quickly frozen for different analyses. The flow chart of the mice treatment procedure is depicted in Figure 1A.

### 2.4 Oral glucose tolerance test (OGTT), insulin tolerance test (ITT), and pyruvate tolerance test (PTT)

The OGTT, ITT, and PTT were conducted during weeks 6 and 7. The mice were fasted 10 h before OGTT and then glucose (2 g/kg) was administered through gavage. Thereafter, blood glucose levels of all the mice were measured at 30, 60, 90, and 120 min after glucose loading. The mice were fasted for 6 h before ITT and were intraperitoneally injected with 0.75 IU/kg insulin. Afterwards, blood glucose levels of all the mice were measured at 30, 60, 90, and 120 min after insulin injection. The mice were fasted 10 h before PTT and were given pyruvate (1.5 g/kg) through gavage. Thereafter, the blood glucose levels of all the mice were measured at 30, 60, 90, and 120 min after pyruvate loading. The area under the curve (AUC) was calculated.

### 2.5 Biochemical analysis

The serum LDL-C, HDL-C, TC, TG, ALT, AST and insulin levels were assayed using commercial detection kits in accordance with the manufacturer’s instructions. The homeostasis model assessment-insulin resistance (HOMA-IR) and insulin sensitivity index (ISI) were calculated according to the following formulas: HOMA-IR = Insulin concentration (ng/mL) × FBG (mmol/L)/22.5, ISI = 1/[FBG (mmol/L) × fasting serum insulin (ng/mL)].

### 2.6 Histopathological analysis

After the fresh liver tissue samples were fixed for 24 h, they were embedded in paraffin and cut into 5 μm thick slices. Following the manufacturer’s instructions, the slices were stained with hematoxylin and eosin (H&E), as well as periodic acid-Schiff staining (PAS) and oil red staining. Finally, all slices were scanned under a scanning microscope (Leica, Aperio CS2, United States) and analyzed.

### 2.7 Immunofluorescence assay

After the mice were sacrificed, the pancreas was harvested, and fixed in 10% formalin, embedded in paraffin and cut into 5 μm sections, to make slides that were subsequently stained with anti-insulin and glucagon antibodies, in the same manner as previously described (Li et al., 2017). Finally, the ImageJ software was used to calculate the fluorescence intensity of insulin and glucagon.

### 2.8 Immunohistochemistry assay

After the mice were sacrificed, a portion of the liver and ileum was removed, fixed in 4% paraformaldehyde for 24 h, embedded in paraffin, and cut into 5-μm-thick sections using a microtome, followed by staining with the relevant antibodies (Zhong et al., 2019). The images were captured under a scanner and the staining positive area were calculated with ImageJ.

### 2.9 Western blotting analysis

A certain mass of tissue was weighed, and the RIPA lysis buffer containing protease inhibitor was added. After the total proteins were extracted, its concentration was determined using the BCA method. Then, the protein concentrations of all the samples were adjusted to the same concentration, and 5 × loading buffer was added. Afterward, the samples were denatured in boiling water at 100°C for 10 min. An equal amount of the denatured proteins was separated using sodium dodecyl sulfate-polyacrylamide gel electrophoresis and transferred onto the PVDF membrane. After blocking with 5% skimmed milk powder for 2 h at room temperature, the membranes were incubated with the corresponding antibodies at 4°C, overnight. After washing thrice, the samples were incubated with the second antibody coupled with horseradish peroxidase for 2 h at room temperature. After washing, the bands were observed using an ECL luminescent reagent under a chemiluminescent system. Then, the protein band densities were analyzed using Image Lab 3.0 software.

### 2.10 Statistical analysis

Data are presented herein as mean ± standard error of the mean (SEM). We analyzed the data using one-way analysis of variance along with Bonferroni’s correction. Differences with a *p* < 0.05 were considered to indicate statistical significance.

## 3 Results

### 3.1 GPS attenuated metabolic disorders in HFD and STZ-induced diabetic mice

To explore the effect of GPS on the metabolism of diabetic mice, the weight, food intake, water consumption, and blood glucose level of all the mice were recorded once a week. The mice that were fed a normal diet steadily gained weight during the study, while the body weight of the HFD mice increased obviously from the first week to the 10th week, and was higher than that of the Nor group. Following STZ injection, the body weight of the HFD mice decreased significantly, and was observably lower than that of the Nor group. During the administration, the results showed that GPS had no effect on the body weight of the diabetic mice (Figure 1A). The RBG, FBG, HbA1c, food intake, and water intake of mice were markedly increased in the Mod group compared with the Nor group (Figures 1B–F), indicating that the metabolism of mice was disordered in the former group. Relative to the Mod group, GPS markedly decreased the RBG of the diabetic mice from the second week (Figure 1B), significantly decreased the FBG of diabetic mice from the first week (Figure 1C), and decreased the HbA1c (Figure 1D), food consumption (Figure 1E), and water consumption (Figure 1F) of the diabetic mice. All in all, these findings indicate that GPS significantly improves metabolic disorders in diabetic mice.

### 3.2 GPS ameliorated glucose homeostasis in HFD and STZ-induced diabetic mice

**FIGURE 2:**
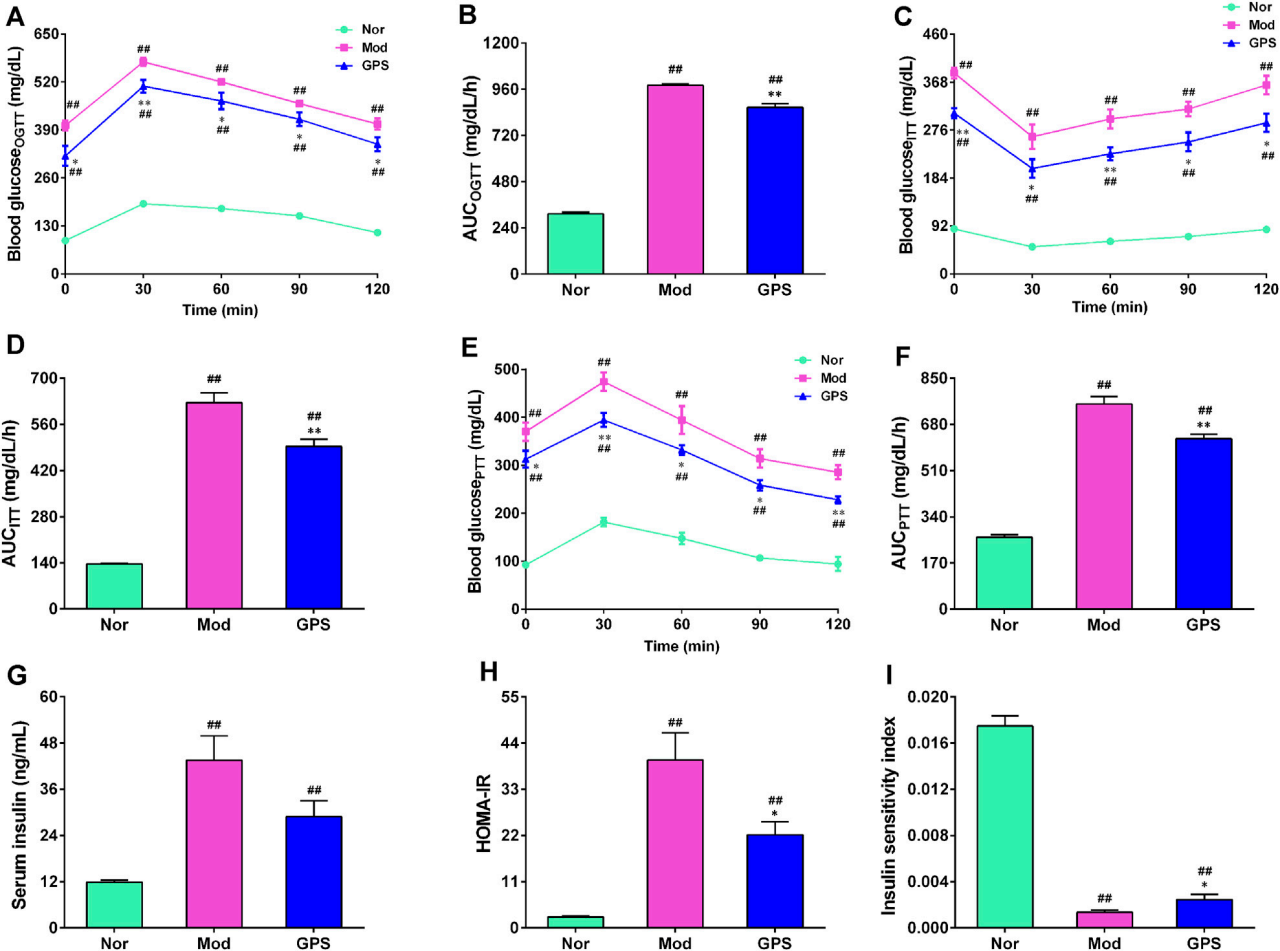
GPS ameliorated glucose homeostasis in HFD and STZ-induced diabetic mice **(A)** OGTT in mice **(B)** AUC of OGTT in mice **(C)** ITT in mice **(D)** AUC of ITT in mice **(E)** PTT in mice **(F)** AUC of PTT in mice **(G)** Serum insulin **(H)** HOMA-IR in mice **(I)** Insulin sensitivity index (ISI) in mice. Data are presented as mean ± SEM, *n* = 10. ^*#*^ *p* < 0.05; ^*##*^ *p* < 0.01 vs. Nor group; *p < 0.05; **p < 0.01 vs. Mod group.

To further investigate the effect of GPS on glucose metabolism, OGTT, ITT, and PTT experiments were conducted during the study. As anticipated, the results of the OGTT and ITT experiments showed that blood glucose at each time point and the AUC of the diabetic mice in the Mod group were remarkably higher, compared with the Nor group (Figures 2A–D), indicating that the diabetic mice had developed abnormal glucose tolerance and insulin resistance. GPS supplementation significantly decreased blood glucose levels at each time point and the AUC of diabetic mice during the two tests (Figures 2A–D). To determine whether the improvement of glucose homeostasis exerted by GPS was associated with gluconeogenesis, we immediately performed a pyruvate tolerance test. The results demonstrated that GPS supplementation resulted in significantly lower blood glucose levels following pyruvate injection (Figure 2E), as well as a significantly smaller AUC (Figure 2F), relative to the Mod group. Next, the insulin content of the serum was measured, and the HOMA-IR and ISI indexes were calculated. The data demonstrated that GPS decreased the serum insulin content of the diabetic mice, but there was no statistical difference (Figure 2G). In addition, GPS also significantly decreased the HOMA-IR index and increased ISI index (Figures 2H,I). Taken together, these results indicate that GPS significantly alleviated glucose metabolism disorders in HFD and STZ-induced diabetic mice.

### 3.3 GPS attenuated islet morphology in HFD and STZ-induced diabetic mice

**FIGURE 3:**
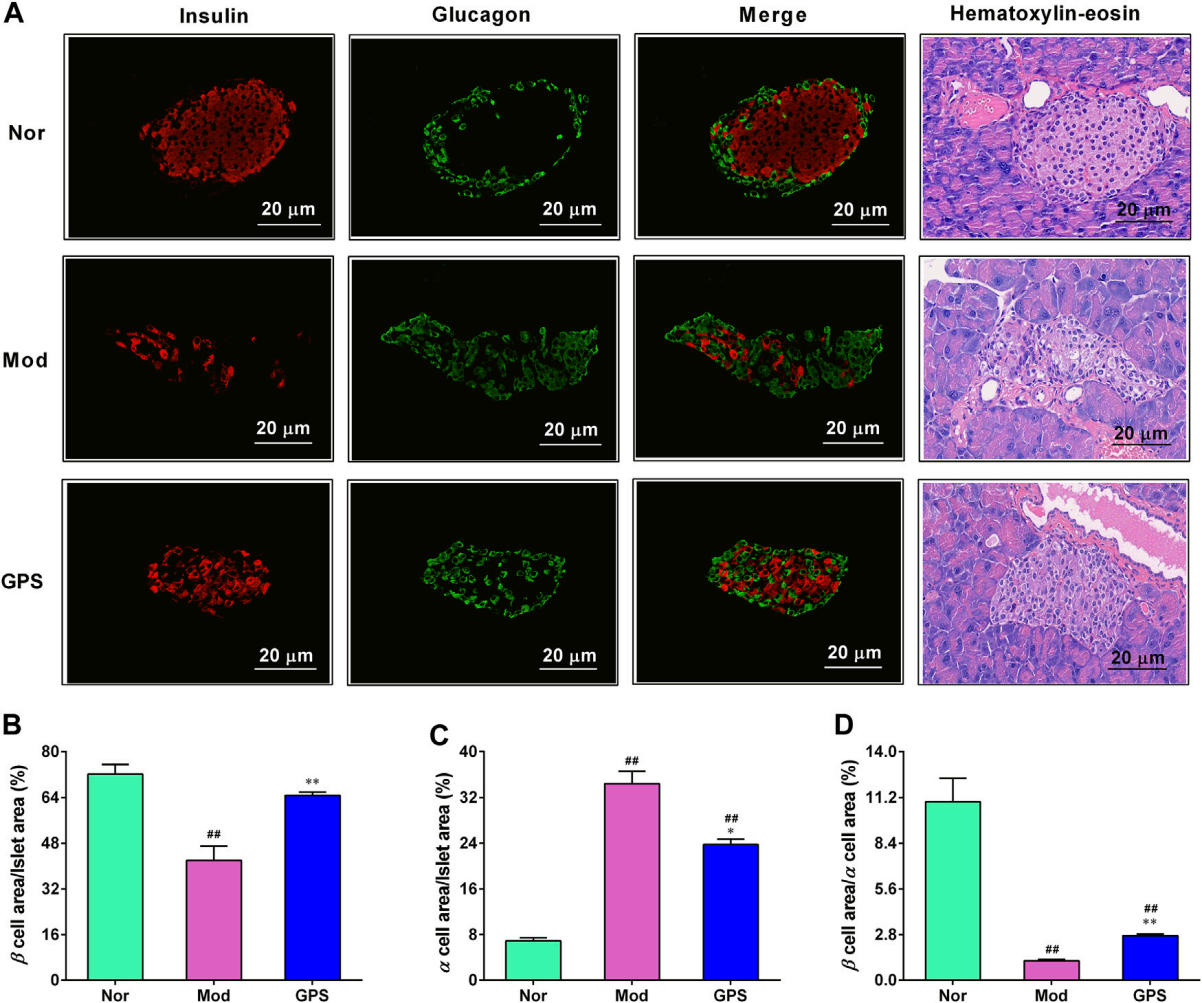
GPS attenuated islet morphology in HFD and STZ-induced diabetic mice **(A)** Representative images of insulin (red) and glucagon (green) immunofluorescence staining of the pancreatic islets. Magnification of all images is at × 200 **(B)** Insulin-positive area to the total islet area **(C)** Glucagon-positive area to the total islet area **(D)** The ratio of insulin to the glucagon. Data are presented as mean ± SEM, *n* = 4. ^*#*^ *p* < 0.05; ^*##*^ *p* < 0.01 vs. Nor group; *p < 0.05; **p < 0.01 vs. Mod group.

To ascertain whether GPS can protect the islets of diabetic mice from hyperglycemia, immunofluorescence staining and HE staining were performed on the islets. As illustrated in Figure 3, relative to the Nor group, the insulin content in the islets of the diabetic mice in Mod group were significantly downregulate, while the glucagon content were markedly upregulated. However, after 7 weeks of GPS treatment, these abnormal changes had been discernibly reversed (Figure 3A). At the same time, the HE staining results also showed that GPS could significantly improve hyperglycemia-induced pancreatic islet injury (Figure 3A). The statistical results of immunofluorescence staining intensity showed that the ratio of the insulin positive area to the islet area was decreased remarkably, while the ratio of the glucagon positive area to the pancreatic islet area was increased remarkably, and the mean fluorescence intensity ratio of insulin to glucagon in the diabetic mice of the Mod group was remarkably decreased, relative to the Nor group mice, indicating an imbalance between α-cells and β-cells. However, these abnormal changes were significantly alleviated following GPS treatment (Figures 3B–D). In summary, these data indicate that GPS plays a pivotal role in maintaining insulin-glucagon homeostasis and improving islet function in HFD and STZ-induced diabetic mice.

### 3.4 GPS ameliorated dyslipidemia in HFD and STZ-induced diabetic mice

**FIGURE 4:**
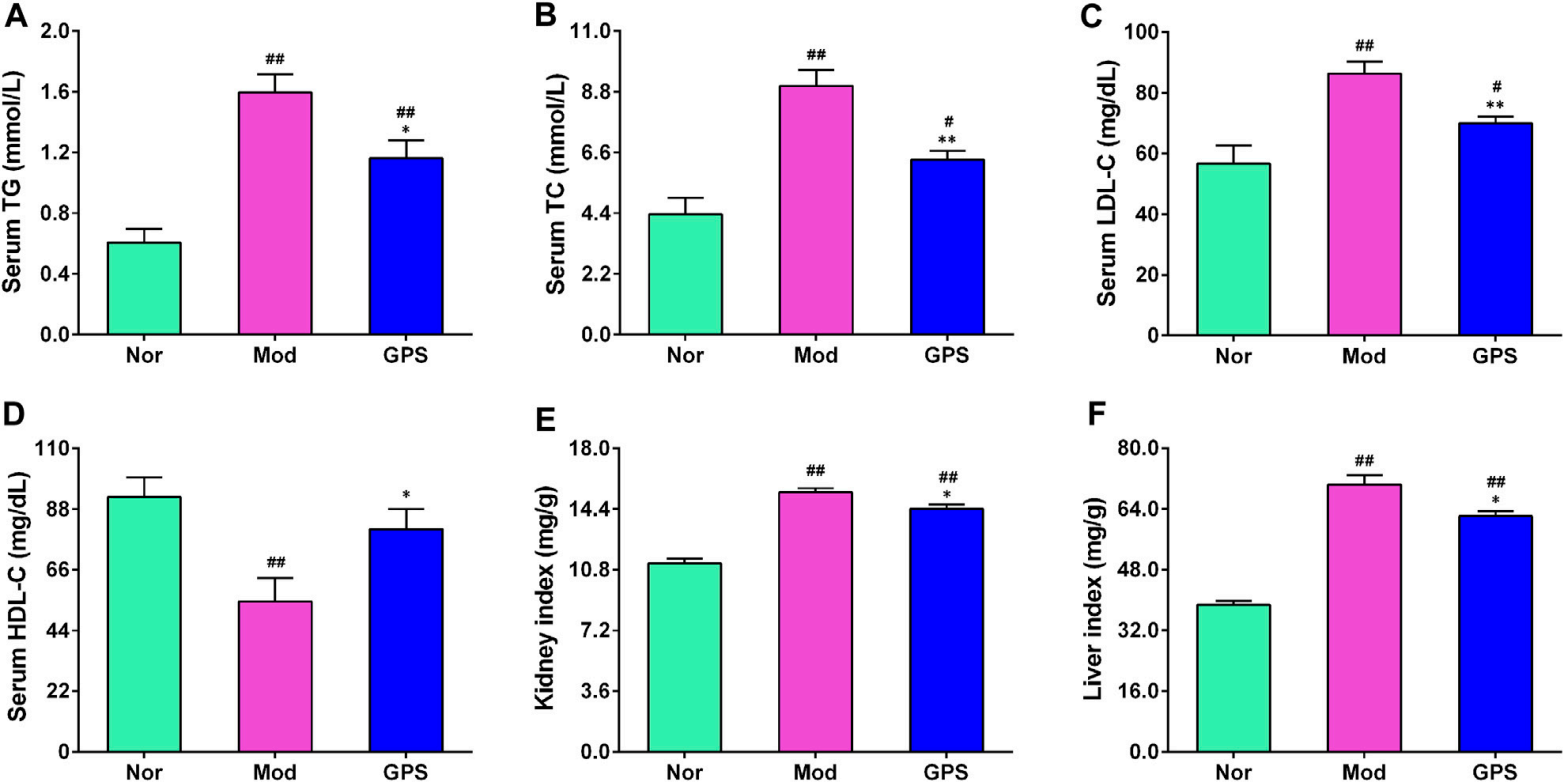
GPS ameliorated dyslipidemia in HFD and STZ-induced diabetic mice **(A)** Serum TG levels **(B)** Serum TC levels **(C)** Serum HDL-C levels **(D)** Serum LDL-C levels **(E)** Kidney index **(F)** Liver index. Data are presented as mean ± SEM, *n* = 10. ^*#*^ *p* < 0.05; ^*##*^ *p* < 0.01 vs. Nor group; *p < 0.05; **p < 0.01 vs. Mod group.

Dyslipidemia is closely associated with the occurrence and development of T2DM (Goldberg et al., 2022). Therefore, the indexes of lipid metabolism in the serum were determined. As illustrated in Figure 4, relative to the Nor group, the serum TG, TC, and LDL-C contents of the diabetic mice in the Mod group were remarkably increased (Figures 4A,B,D), while the HDL-C content was significantly decreased (Figure 4C), indicating that the diabetic mice suffered from observable lipid disorders. However, following GPS treatment for 7 weeks, the serum TG, TC, and LDL-C contents of the diabetic mice decreased significantly (Figures 4A,B,D), and the HDL-C content increased remarkably, compared with the Mod group (Figure 4C). Notably, compared with the Mod group, GPS significantly reduced the kidney index and liver index of the diabetic mice (Figures 4E,F). These results suggest that the amelioration of diabetic symptoms as a result of GPS treatment may be associated with the improvement in blood lipid disorders.

### 3.5 GPS mitigated hepatic steatosis in HFD and STZ-induced diabetic mice

**FIGURE 5:**
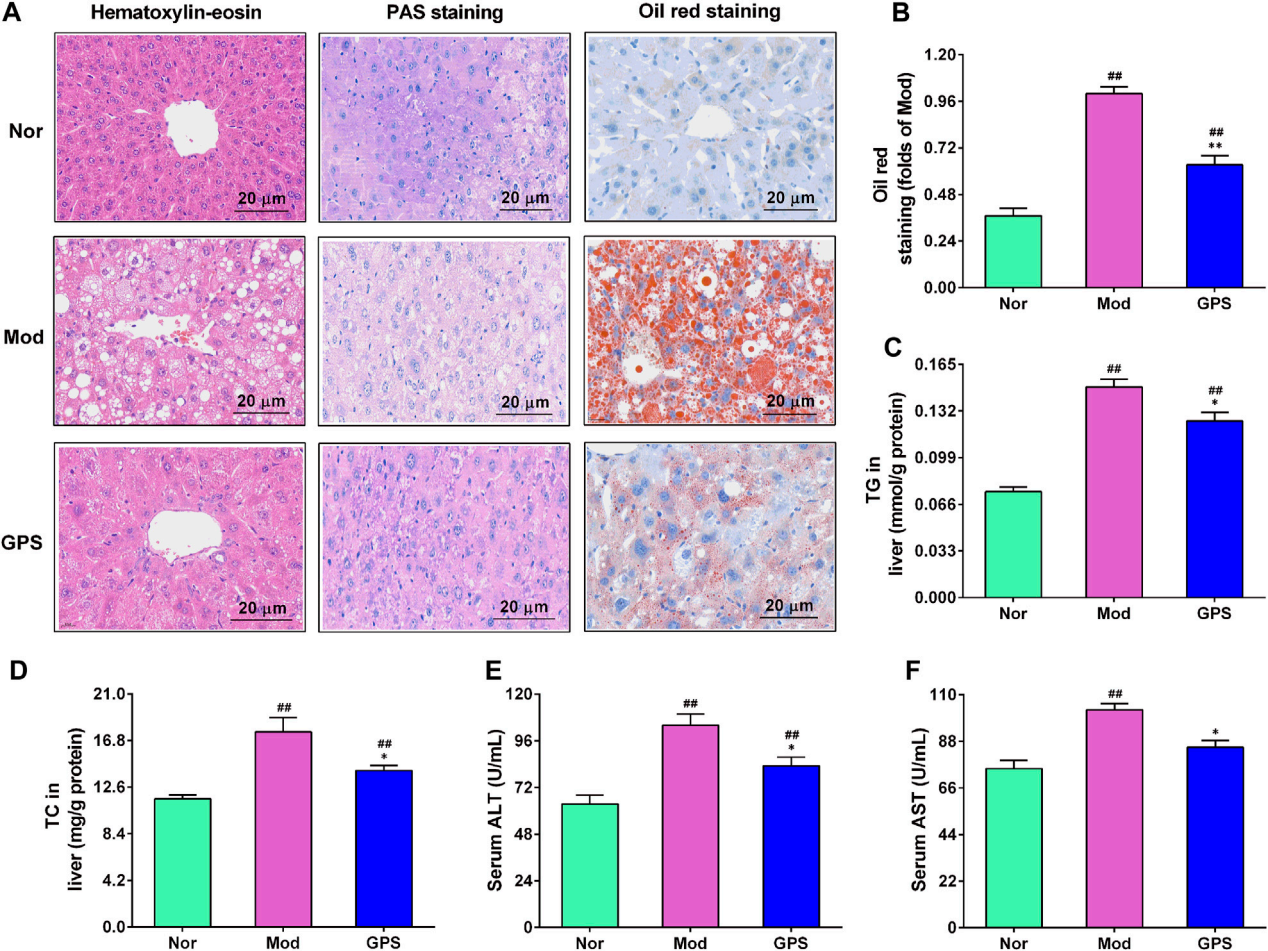
GPS mitigated hepatic steatosis in HFD and STZ-induced diabetic mice **(A)** Representative images of hematoxylin-eosin (HE), periodic acid-Schiff (PAS), and oil red staining of the liver of the diabetic mice, *n* = 4. Magnification of all images were at ×200 **(B)** Statistical analysis of oil red level, *n* = 4 **(C)** TG in the liver, *n* = 10 **(D)** TC in the liver, *n* = 10 **(E)** Serum ALT activities. *N* = 10 **(F)** Serum AST activities, *n* = 10. Data are presented as mean ± SEM. ^*#*^ *p* < 0.05; ^*##*^ *p* < 0.01 vs. Nor group; *p < 0.05; **p < 0.01 vs. Mod group.

Hepatic steatosis plays a fundamental role in the occurrence and development of type 2 diabetes Lee et al., 2022a. Therefore, HE staining, PAS staining, and oil red staining were performed on the liver. As illustrated in Figure 5A, the results of the H&E staining demonstrated that, relative to the Nor group, hepatocytes of the Mod group were filled with many vacuolated round lipid droplets of different sizes, and that the hepatocytes were significantly swollen, damaged, disorganized, and infiltrated by inflammatory cells. After 7 weeks of treatment with GPS, the abnormal changes in the liver of diabetic mice were found to have improved evidently (Figure 5A). The results of PAS staining also showed that GPS significantly improved structural and morphological abnormalities, and increased glycogen accumulation in the liver of the diabetic mice (Figure 5A). The results of the oil red staining revealed that GPS treatment could remarkably decrease liver fat accumulation in diabetic mice (Figure 5A), and the quantitative analysis results of ImageJ further confirm this result (Figure 5B). Furthermore, GPS treatment also remarkably decreased the TG and TC levels in the liver of the diabetic mice (Figures 5C, D), and significantly weakened ALT and AST activities in the serum (Figures 5E, F). Together, the above results suggest that the improvement of type 2 diabetes symptoms caused by GPS might be associated with the decrease in hepatic steatosis in mice.

### 3.6 GPS inhibited hepatic gluconeogenesis by regulating the PI3K/AKT/FOXO1 signaling pathway in HFD and STZ-induced diabetic mice

**FIGURE 6:**
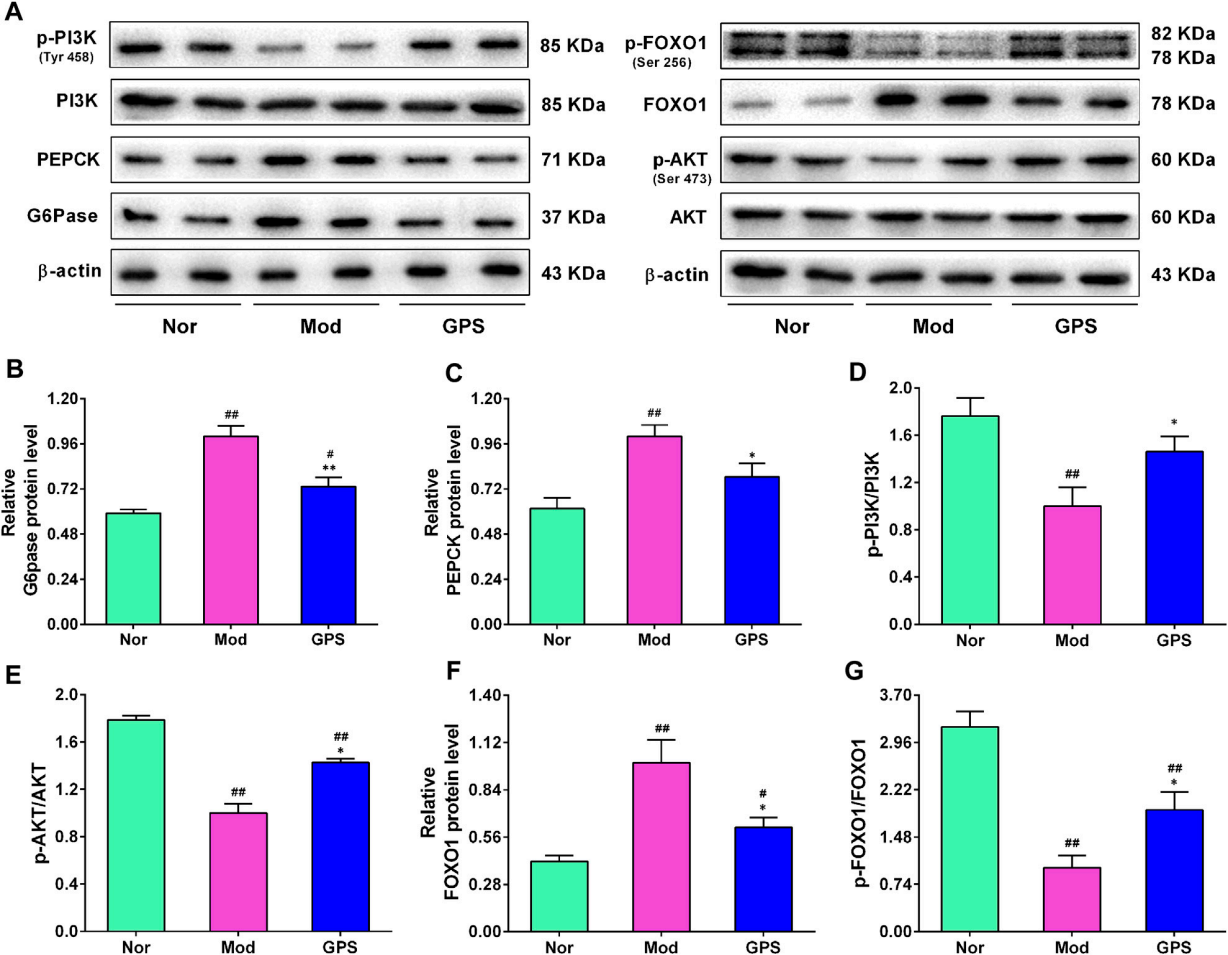
GPS inhibited hepatic gluconeogenesis by regulating the PI3K/AKT/FOXO1 signaling pathway in HFD and STZ-induced diabetic mice **(A)** Representative Western blotting analysis results of G6Pase, PEPCK, PI3K, p-PI3K, AKT, p-AKT, FOXO1, and p-FOXO1 **(B)** Relative optical density of G6Pase levels **(C)** Relative optical density of PEPCK levels **(D)** The ratio of p-PI3K/PI3K **(E)** The ratio of p-AKT/AKT **(F)** Relative optical density of FOXO1 expression **(G)** The ratio of p-FOXO1/FOXO1. Data are presented as mean ± SEM, *n* = 6. ^*#*^ *p* < 0.05; ^*##*^ *p* < 0.01 vs. Nor group; *p < 0.05; **p < 0.01 vs. Mod group.

To investigate whether GPS-induced improvements of the symptoms of type 2 diabetes were associated with the inhibition of gluconeogenesis, the expression levels of PEPCK and G6Pase were evaluated using immunohistochemistry and Western blotting assays. As shown in Figures 6A–C, the results of the Western blotting analysis illustrated that the protein expression levels of PEPCK and G6Pase in the liver of the Mod group mice were remarkably elevated, compared with the Nor group, and that 50 mg/kg GPS significantly suppressed the expression of PEPCK and G6Pase. The PI3K/AKT/FOXO1 signaling pathway is closely associated with gluconeogenesis. To evaluate whether the GPS-induced gluconeogenesis inhibition was associated with this signaling pathway, Western blotting analysis was performed to detect the expression levels of associated proteins in the liver of the diabetic mice. As illustrated in Figure 6A–G, relative to the Nor group mice, the protein levels of p-PI3K, p-AKT, and p-FOXO1 were remarkably downregulated, along with the upregulation of FOXO1 expression in liver of the Mod group mice. However, after 7 weeks of GPS treatment, the abnormal changes associated with this signaling pathway-related protein in liver of the diabetic mice had improved significantly, relative to the Mod group (Figure 6A–G). Collectively, all these data indicate that GPS significantly inhibits gluconeogenesis by regulating the PI3K/AKT/FOXO1 signaling pathway in liver of the diabetic mice, indicating that this may be the potential mechanism by which GPS treatment improves symptoms of type 2 diabetes.

### 3.7 GPS enhanced the intestinal integrity in HFD and STZ-induced diabetic mice

**FIGURE 7:**
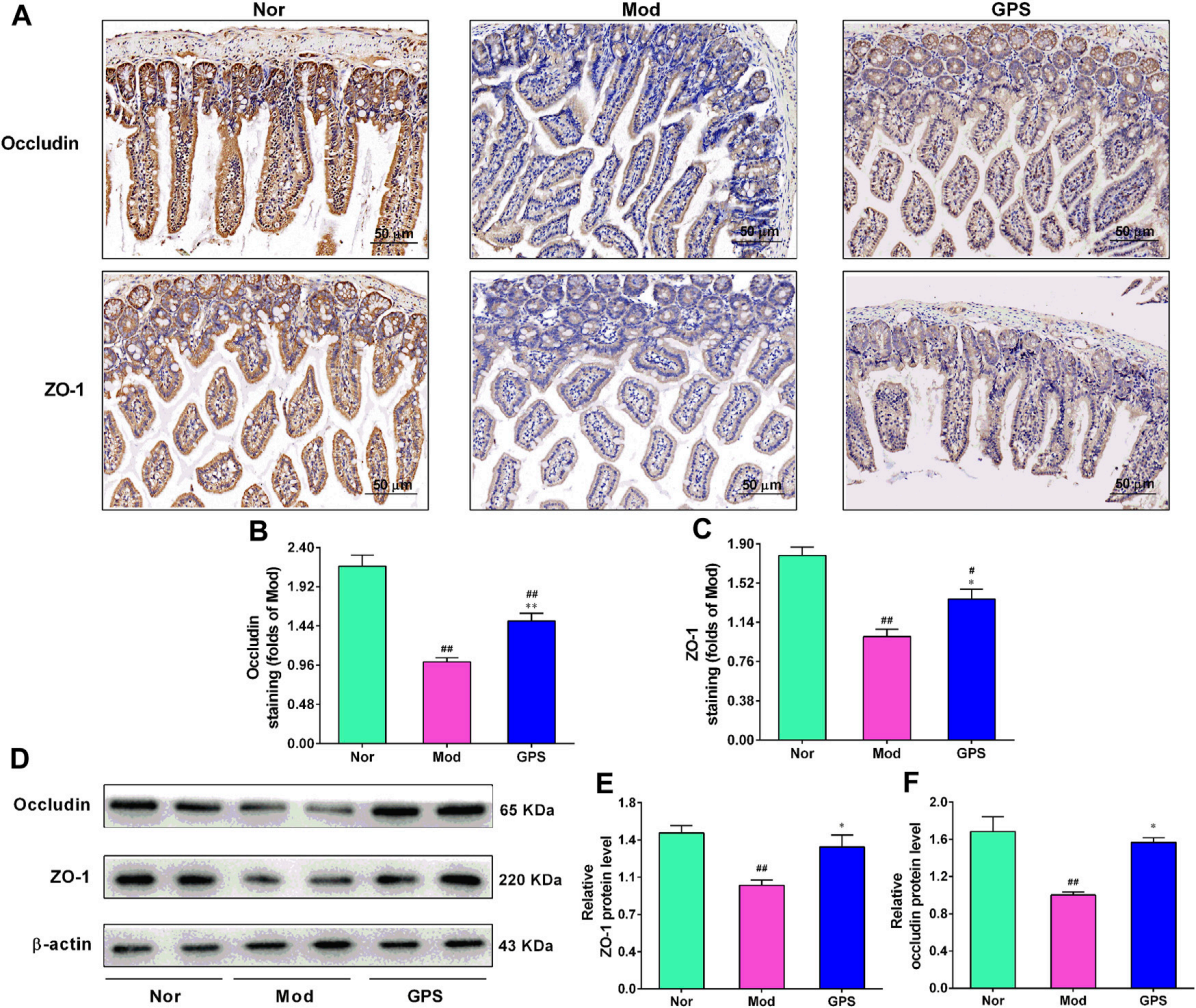
GPS enhanced intestinal integrity in HFD and STZ-induced diabetic mice **(A)** Representative images of immunohistochemical staining performed to determine ZO-1 and occludin expression levels in the ileum of the diabetic mice, *n* = 4. The magnification of all images is at × 200 **(B)** Statistical analysis of occluding protein expression, *n* = 4 **(C)** Statistical analysis of ZO-1 protein expression, *n* = 4 **(D)** Representative protein bands of ZO-1 and occludin, *n* = 6 **(E)** Relative optical density of ZO-1 levels, *n* = 6 **(F)** Relative optical density of occludin levels, *n* = 6. Data are presented as mean ± SEM. ^*#*^ *p* < 0.05; ^*##*^ *p* < 0.01 vs. Nor group; *p < 0.05; **p < 0.01 vs. Mod group.

To study whether GPS is beneficial for the intestinal barrier function of type 2 diabetic mice, the ileum of the mice were analyzed using immunohistochemistry. As illustrated in Figures 7A–C, relative to the Nor group, positive staining of ZO-1 and occludin in ileum of the Mod group were lighter. However, after 7 weeks of GPS treatment, the positive staining of these two indicators became darker, compared with the Mod group. To further verify the immunohistochemistry results, the protein expression of ZO-1 and occludin in the ileum were evaluated using Western blotting. The results demonstrated that GPS remarkably increased the protein expression of ZO-1 and occludin in the ileum of the diabetic mice, relative to the Mod group (Figures 7D–F), in agreement with results of the immunohistochemistry. Taken together, all these data indicate that GPS can help maintain the integrity of the intestinal barrier in type 2 diabetic mice.

## 4 Discussion

T2DM is chiefly characterized by the decrease in insulin secretion or decreased sensitivity of target organs to insulin, which in turn leads to hyperglycemia, hyperlipidemia, and the increase in glucose production in the liver (Dao et al., 2023). Its typical clinical manifestations are polydipsia, polyphagia, polyuria, and weight loss. T2DM and its complications are one of the principal causes of morbidity and mortality worldwide and are currently on the rise, and have seriously affected the quality of life and imposed a very heavy economic burden on society (Faselis et al., 2020). At present, the main methods of treatment used for T2DM are insulin injection and the oral administration of one or more hypoglycemic agents (Ke et al., 2022). Although wide range of drugs with certain curative effects are available, the number of patients with T2DM continues to increase annually. Owing to the ineffective control of T2DM, new treatment strategies are increasingly needed. GPS can improve liver diseases, such as alcoholic liver disease (Li et al., 2018; Yang et al., 2020), cholestatic liver disease (Tang et al., 2016), and drug-induced liver injury (Han et al., 2018). In addition, other studies have found that GPS can alleviate diabetes (Xiao et al., 2022), diabetic retinopathy (Zhang et al., 2019), diabetic nephropathy (Xiao et al., 2020), and peripheral neuropathy (Lu et al., 2018). However, the metabolic pathway through which GPS improves T2DM and gluconeogenesis, and regulates gluconeogenesis remains has not been elucidated as yet. At present, the HFD/STZ-induced T2DM model had been widely used to study the activity and mechanism of various drugs for diabetes. In this study, we explored the potential effects and mechanism by which GPS treatment acts on gluconeogenesis in type 2 diabetic mice, and determined whether the inhibition of GPS during gluconeogenesis can be mediated by regulating the PI3K/AKT/FOXO1 signaling pathway.

It is well recognized that HFD combined with a low-dose STZ injection is sufficient to establish a model of insulin resistance with typical pathological features of T2DM (Srinivasan et al., 2005). In our study, the RBG, FBG, HbA1c, food intake, and water consumption of mice in the Mod group were found to be markedly upregulated than those in the Nor group, while the body weight of the mice was remarkably downregulated, relative to the Nor group following the injection of STZ. These characteristics are consistent with that of clinically diabetic patients. These characteristics may be a result of impaired energy metabolism and insufficient energy supply caused by body’s insufficient utilization of glucose, which leads to the increase in food and water consumption in the diabetic mice. In addition, abnormal glucose metabolism also leads to excessive consumption of muscle, fat, and protein tissues, which in turn leads to weight loss and a further increase in FBG. However, GPS treatment could discernibly improve these abnormal changes, which is in accordance with the results of previous studies (Xiao et al., 2022). Furthermore, the sensitivity of the liver, muscle, and fat of T2DM patients to insulin is reduced, and the ability of the body to handle glucose is also significantly reduced, indicating obvious signs of insulin resistance and abnormal glucose tolerance (Lee et al., 2022b). On the other hand, long-term hyperglycemia caused by the overloading of the pancreas, resulting in the secretion of an excessive quantity of insulin to maintain blood sugar, which leads to impaired pancreatic function and abnormal islet structure (Wolpin et al., 2013). To further study the effects of GPS on glucose metabolism and the pancreatic morphology in mice with type 2 diabetes, we determined the levels of the relevant indicators. The results showed that OGTT, ITT, and the pancreatic morphology of the mice in the GPS group had improved remarkably. These results indicate that GPS exerts a potential therapeutic effect on type 2 diabetes induced by HFD/STZ.

Dyslipidemia is very common among T2DM patients. It is usually one of the early manifestations of insulin resistance (IR), sometimes even before the diagnosis of diabetes. Therefore, IR in type 2 diabetes is relative to dyslipidemia (Bahiru et al., 2021). Moreover, IR and insulin secretion deficiency are the main causes of dyslipidemia in type 2 diabetes. When IR occurs, the synthesis of lipoprotein lipase in the body is reduced, which in turn reduces the hydrolysis of TG, which leads to lipid metabolism disorders (Kim and Song, 2022). In addition, high levels of TG require a higher level of insulin to compensate for the decrease in insulin sensitivity, which further aggravates IR, leading to a vicious circle (Fan et al., 2018). In this study, we observed a significant improvement in blood lipid disorders in the mice treated with GPS. The liver is a central organ for regulating lipid metabolism and maintaining energy homeostasis. If the lipid content exceeds the metabolic capacity of the liver, lipid is stored in the liver in the form of lipid droplets, which may cause long-term liver inflammation, insulin resistance, and diabetes (Heeren and Scheja, 2021). However, persistent hyperglycemia promotes abnormal TG deposition in the liver cells, which further promotes liver steatosis (Zhang et al., 2018). Therefore, we also investigated the effect of GPS treatment on liver steatosis and found that GPS could significantly slow down lipid accumulation in the liver of the type 2 diabetic mice. However, we failed to investigate the mechanism through which GPS improves hepatic steatosis and will be examined through further research in the future.

The pathophysiological mechanism that leads to elevated blood glucose involves a variety of tissues and cells, as the liver is very closely associated with T2DM. Among these mechanisms, the increase in the production of endogenous glucose through gluconeogenesis is regarded as one of the main causes of high FBG in T2DM (Liu et al., 2021). At present, a large number of studies have shown that inhibition of liver gluconeogenesis is effective in reducing blood glucose, which is also a reliable direction for the development of anti-diabetic drugs (Petersen et al., 2017). During fasting, blood glucose is mainly derived from gluconeogenesis and glycogen decomposition, while in patients with T2DM with long-term fasting, blood glucose is mainly derived from gluconeogenesis, indicating that gluconeogenesis plays a crucial role in the development of T2DM (Rizza, 2010). In our PTT experiment, the gluconeogenesis of mice in the Mod group increased significantly, while GPS treatment could significantly inhibit gluconeogenesis. Therefore, there is a need to better understand the molecular mechanism underlying the inhibition of gluconeogenesis induced by GPS. The PI3K/Akt pathway is one of the most important signal transduction pathways that operate under the action of insulin (Huang et al., 2018). The PI3K signaling molecule regulates glucose uptake and metabolism by regulating glycogen synthesis and gluconeogenesis, while dysfunctional PI3K leads to an imbalance in glucose metabolism (Zhu et al., 2023). AKT is a direct downstream molecule regulated by PI3K. It is also a key enzyme for regulating glucose metabolism. AKT can be directly activated by insulin to mediate various biological effects associated with glucose metabolism (Sun et al., 2019). FOXO1 is a transcription factor that plays a fundamental role in the regulation of HGP and insulin sensitivity. Studies have shown that elevated FOXO1 expression in the liver may lead to an increase in FBG in mice (Van Berendoncks et al., 2015), while the inhibition of FOXO1 expression in the liver may delay the progression of diabetes and fatty liver (Herder et al., 2016). G6Pase and PEPCK are rate-limiting enzymes that are involved in gluconeogenesis (Zhu et al., 2021). PEPCK catalyzes oxaloacetate to phosphoenolpyruvate, and then converts it to glucose. The function of G6Pase is to hydrolyze glucose 6 phosphate into glucose and inorganic phosphate, and the glucose is finally transported by transporters (Sun et al., 2021). In T2DM, PI3K/Akt activity is inhibited, and the gene expression of gluconeogenesis-related genes (PEPCK and G6Pase), is enhanced by FOXO1. In this study, GPS treatment was shown to increase the expression of p-PI3K, p-AKT, and p-FOXO1 at protein level, and also to inhibit the expression of PEPCK and G6Pase at protein level in the liver of diabetic mice, indicating that GPS treatment may be a viable manner of improving IR and hyperglycemia by regulating the PI3K/AKT/FOXO1 signaling pathway to inhibit gluconeogenesis.

T2DM and intestinal permeability interact with each other. On the one hand, the ecological imbalance of intestinal microflora and T2DM will lead to the impairment of intestinal permeability (Rehman et al., 2022). On the other hand, the increased intestinal permeability will promote the progress of T2DM (Cao et al., 2020). Moreover, as important structural proteins with tight junction, ZO-1 and occludin play important roles in maintaining intestinal permeability and regulating intestinal barrier function (Sharma and Tripathi, 2019). ZO-1 and occludin block proteins and cytoskeletons by connecting tight junction proteins. When abnormally expressed, these will damage the intestinal mechanical barrier and promote the occurrence and development of diseases (König et al., 2016). Furthermore, it has also been found that T2DM is usually accompanied by increased intestinal permeability, as evidenced by decreased protein of tight junction (Xu et al., 2019; Cao et al., 2020; Rehman et al., 2022). Herein, we found that GPS significantly increased the expression of protein ZO-1 and occludin in the ileum of diabetic mice, indicating that GPS may have an impact on intestinal flora of diabetic mice. However, we have not deeply studied the reason why GPS reduces intestinal permeability. Therefore, future study will thus be focused on clarification of the echanism of GPS in improving T2DM from intestinal permeability.

There remain certain limitations for our research. Firstly, the effects of GPS on the proteins and genes related to insulin resistance and fat accumulation in the liver of the HFD and STZ-induced diabetic mice were not studied, which made the research in this field lacking. Secondly, muscle and adipose tissue are the target organs of insulin and also play a pivotal role in maintaining glucose homeostasis. However, we did not investigate the effects of GPS on these two organs. Thirdly, the effect of GPS on gluconeogenesis and the possible mechanism were not studied in the corresponding cell model. In addition, when the research was carried out on HFD and STZ-induced diabetic mice, there were no positive control drugs and more dose groups, and the effects of GPS on all aspects of normal mice at such dose were not investigated. It would be more convincing and more meaningful if supported by these relevant data. Lastly, we found that GPS played a certain role in improving the intestinal barrier, but we did not explore the possible mechanisms including the intestinal flora and intestinal microecology. Nevertheless, further investigation of DPDS is highly desirable because it has many known pharmacological effects and unknown pharmacological effects that warrant further investigation. Moreover, further research is needed to better understand the protective mechanism of GPS for T2DM.

**FIGURE 8:**
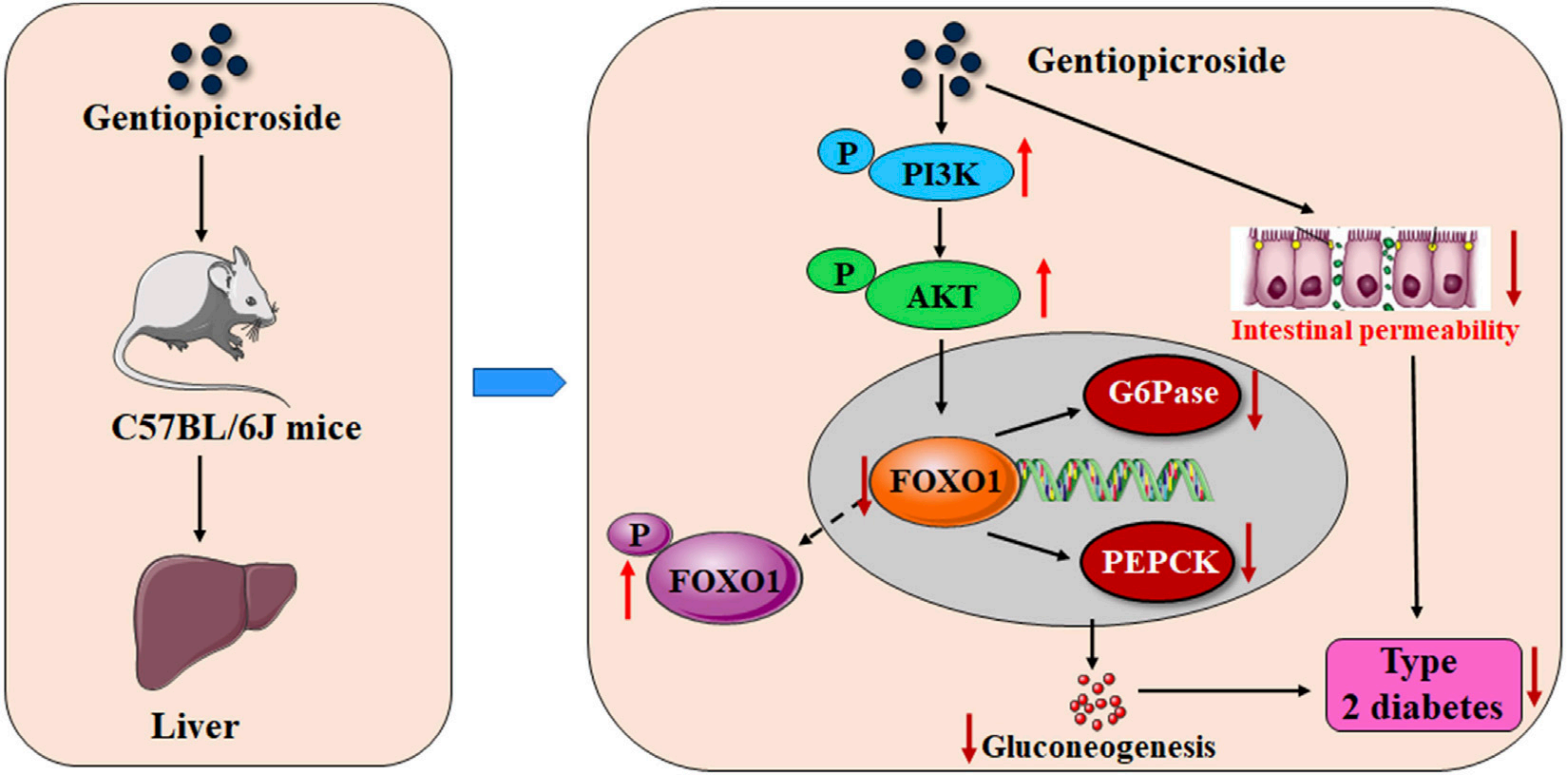
Schematic representation indicating the potential mechanisms of GPS against T2DM.

In summary, our findings clearly indicated that GPS treatment can improve symptoms of the type 2 diabetic mice to a certain extent. The underlying mechanism of action involved may be that GPS induces the enhancement of the phosphorylation of FOXO1 by activating PI3K/AKT in the liver of diabetic mice, and to further inhibit the expression levels of G6Pase and PEPCK, thus inhibiting gluconeogenesis, and may also be related to the enhancement of intestinal integrity (Figure 8). The findings of this study provide a direct experimental basis for the role and potential mechanism by which GPS exerts its anti-T2DM effects. Although GPS has been found to have a variety of pharmacological effects, it is still a long way from clinical application.

## Data Availability

The raw data supporting the conclusion of this article will be made available by the authors, without undue reservation.
